# Integrating Evaluation into Exoskeleton Systems: A Model-Based Approach

**DOI:** 10.3390/s26133971

**Published:** 2026-06-23

**Authors:** Kathy S. Min, Homayoon Kazerooni

**Affiliations:** Department of Mechanical Engineering, University of California, Berkeley, CA 94720, USA; songheemin@berkeley.edu

**Keywords:** exoskeleton, evaluation platform, embedded systems, biomechanical loading, real-time monitoring, occupational safety, human–robot interaction

## Abstract

The evaluation of wearable robotic systems remains a challenge, particularly in real-world environments where laboratory-based methods are impractical. Existing approaches rely on external instrumentation, such as surface electromyography (sEMG) or motion capture, which are difficult to deploy continuously and do not directly measure key internal metrics such as joint loading or spinal forces. This work introduces a new paradigm for exoskeleton evaluation in which biomechanical assessment is embedded directly within the device’s sensing and computational architecture. We present the ExoMetrix system, a platform that integrates onboard sensing, real-time data acquisition, cloud-based processing, and user-facing analytics into a unified workflow for continuous evaluation of human–exoskeleton interaction. Sensor data from the device are streamed and processed using physics-based models. The resulting outputs are translated into estimates of internal biomechanical quantities, including joint torques, spinal compression and shear forces, and muscle loading. By enabling real-time feedback and longitudinal monitoring without external instrumentation, this approach transforms evaluation from an external, episodic process into an embedded and continuous capability, supporting safer and more scalable deployment of exoskeleton technologies.

## 1. Introduction

A fundamental question in wearable robotics is: what is the optimal real-time framework for evaluating an exoskeleton or exosuit worn by a person? These devices interface directly with the human body, exchanging power through physical contact forces that significantly alter the user’s dynamics. While these effects can be mathematically characterized, the specific outcome is highly dependent on the unique mechanical interaction dictated by the device’s kinematic structure (e.g., geometry, joint layout) and dynamic properties (e.g., stiffness, damping, actuation behavior) [1].

Conventional evaluation strategies rely on a “gold-standard” triad of biomechanical instrumentation: surface electromyography (EMG) to monitor muscle activity and fatigue, indirect calorimetry to measure metabolic cost, and optical motion capture (MoCap) to track precise kinematics and posture. Direct measurement techniques such as EMG and kinematic assessments remain the dominant methodologies in occupational exoskeleton evaluation, with most studies relying on laboratory-based protocols. These methods have provided foundational insights into how exoskeletons reduce muscular effort [2,3,4,5], lower the energetic cost of locomotion [6,7,8], and mitigate injury risk through posture modification [9,10,11]. EMG-based assessments have also been applied to shoulder exosuits [12,13].

While invaluable in laboratory settings, methods like EMG face significant challenges in real-world, industrial deployments. Surface sensors require meticulous calibration, are prone to noise and motion artifacts, and are often impractical for continuous, long-term use [14,15,16,17,18,19]. Crucially, sEMG measures muscular response but does not directly quantify internal joint loading or spinal forces—key indicators of biomechanical stress and injury risk.

**Figure 1:**
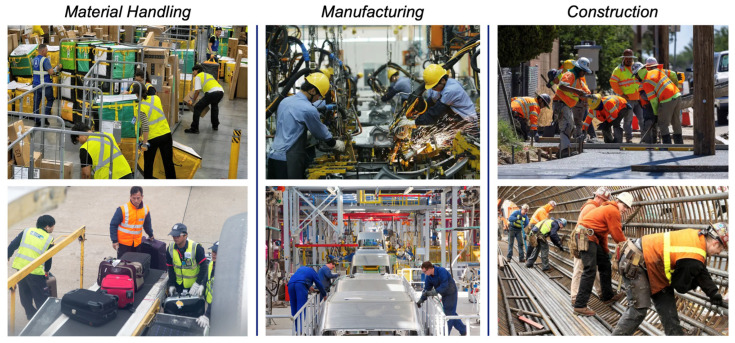
Industrial exoskeletons are deployed in places like warehouses, manufacturing lines, and construction sites—environments that are dynamic and uncontrolled. It is difficult to place motion-capture cameras and metabolic carts on factory floors.

In addition, these laboratory-grade tools are fundamentally ill-suited for the environments where exoskeletons are intended to function. While invaluable for controlled studies, they require specialized equipment, dedicated staff, and extensive calibration, which inherently limits research to small sample sizes and brief, episodic snapshots of performance [20]. Consequently, while we have developed a deep understanding of how exoskeletons behave in the lab, we remain largely blind to their performance in the real world [21,22]. In situ environments—such as warehouses, manufacturing lines, and construction sites shown in Figure 1—are dynamic, noisy, and uncontrolled. One cannot feasibly deploy a metabolic cart on a factory floor or install motion-capture volumes across a sprawling construction site. Our current evaluation tools simply do not scale to the demands of the field, where the challenge is not just measuring one device in a controlled trial, but understanding the performance of hundreds to thousands of units in real time.

To address this gap, this work introduces a new paradigm for exoskeleton evaluation in which biomechanical assessment is embedded directly within the device’s sensing and computational architecture. By leveraging onboard sensors (e.g., encoders, IMUs, torque sensors, and current sensors) in combination with physics-based or data-driven models, the proposed framework enables real-time estimation of internal biomechanical quantities—including joint torques, spinal compression and shear forces, and muscle loading—without reliance on external laboratory instrumentation. In contrast to prior approaches that depend on offline analysis or specialized measurement systems, this integration supports continuous, field-deployable monitoring of human-exoskeleton interaction [20,21].

We have developed such a platform: the ExoMetrix system. This paper presents a prototype for continuous, model-based evaluation that operates as a native function of the exoskeleton system itself. The biomechanical quantities presented in this work should be interpreted as model-based estimates derived from onboard sensing and physics-based calculations. While these metrics provide useful real-time indicators of biomechanical loading, a dedicated validation study remains necessary before they can be considered validated biomechanical or clinical measurements.

Beyond serving as an engineering tool, we posit that embedded self-evaluation is a fundamental requirement for the safe and scalable deployment of wearable robotics. In occupational and clinical settings, exoskeletons can provide transparent, real-time feedback regarding their biomechanical impact to users, supervisors, and clinicians. This feedback, delivered via a user interface or other modalities, may include metrics such as muscle effort and load sharing, joint torque reduction, fatigue estimation, spinal loading, posture deviation, and cumulative exposure over time—enabling longitudinal monitoring, informed decision-making, and improved user trust.

## 2. Further Motivation for an Integrated Evaluation Platform

The development of this platform is motivated by several critical needs that extend beyond pure performance measurement:

Device Personalization: Integrated assessment enables adaptive control strategies that can tailor assistance levels in real-time based on an individual’s biomechanics and task demands.

Longitudinal Health Monitoring: Continuous data collection over time facilitates health surveillance and proactive injury prevention for workers, providing a digital record of physical exposure.

Regulatory Compliance & Standards: Objective, real-time metrics are essential for demonstrating compliance with emerging ergonomic standards (e.g., ASTM F3474 and ASTM F3795 [23,24]) on exoskeleton safety and performance) and for validating device efficacy in reducing injury risk.

User Acceptance and Trust: Transparency regarding the exoskeleton’s impact builds user trust, improves safety perception, and fosters adoption by demystifying the device’s function.

Embedding evaluation capabilities into the exoskeleton closes the feedback loop between device operation and human outcomes. This capability may support safer and more informed use of wearable robotic systems.

## 3. Workflow of ExoMetrix System

**Figure 2:**
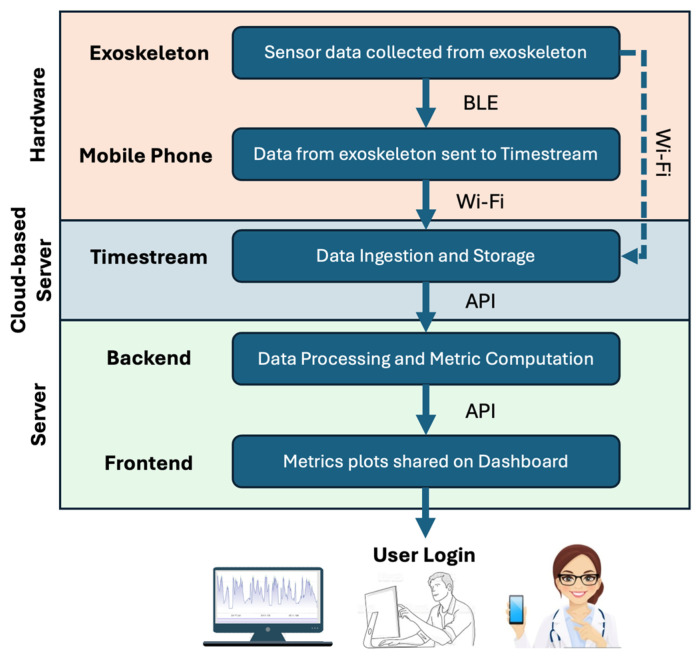
System Architecture of ExoMetrix, a platform for displaying understandable biomechanical metrics based on exoskeleton sensor data.

As shown in Figure 2, ExoMetrix is at a high level composed of Hardware components (the Exoskeleton worn by a worker and their Mobile Phone), a Cloud-based Streaming Server, and a Server, containing a Frontend and Backend Server.

At the hardware level, an exoskeleton’s embedded sensors and its onboard controller are used to capture kinematic and actuation signals during operation. These data streams are transmitted wirelessly using either the exoskeleton’s direct Wi-Fi connectivity or the Bluetooth Low Energy (BLE) of the wearer’s Mobile Phone. In both cases, the transmitted data are collected in a Cloud-based Streaming Server, where they are time-stamped, indexed by the exoskeleton ID, and stored for subsequent retrieval and analysis. This method enables a large number of exoskeletons to send their data to the Cloud-based Streaming Server.

The next element of this platform is a Server that includes a Backend and a Frontend. Using Application Programming Interfaces (APIs), the Cloud-based Streaming Server, the Backend, and the Frontend all communicate with each other [25]. Upon prompting at a manager’s dashboard located in the Frontend Server, the relevant data is read from the Cloud-based Streaming Server. The biomechanical and ergonomic metrics are then computed by the Backend Server, and the results are presented in user-friendly visualizations on the manager’s dashboard in the Frontend Server.

We now go into the specifics of each layer below.

### 3.1. Hardware: Exoskeleton

To enable real-time monitoring and downstream biomechanical analysis, the Exoskeleton is equipped with embedded sensors that capture both kinematic and actuation data [26]. Kinematic data, providing estimates of body posture and limb angles, is captured by inertial measurement units (IMUs) and motor encoders. Actuation data, which measures the mechanical assistance delivered by the exoskeleton, is captured by integrated torque and current sensors within the actuators. Together, these synchronized datasets form the raw data stream used for the subsequent estimation of biomechanical metrics—such as internal muscle forces and joint forces—by the Backend Server. Each measurement is time-stamped and tagged with a unique exoskeleton ID.

### 3.2. Hardware: Mobile Phone

Figure 2 depicts two methods for transmitting the raw data to the Cloud-based Streaming Server. One approach uses an integrated Wi-Fi chipset within the exoskeleton to send data directly to the server. However, in our initial experiments, the exoskeletons lacked integrated Wi-Fi. Therefore, they used Bluetooth Low Energy (BLE) to transmit data to the wearer’s Mobile Phone, which then relayed it to the Cloud-based Streaming Server using the phone’s Wi-Fi connection.

Using a mobile phone as a bridge offers three primary advantages. First, BLE radios have a significantly lower power draw than Wi-Fi or cellular modules, which preserves the exoskeleton’s battery life. Second, this method mitigates connectivity issues in industrial settings. Direct transmission from the exoskeleton requires reliable, ubiquitous Wi-Fi or cellular coverage to prevent data loss, unless the exoskeleton has local storage. In contrast, a mobile phone can buffer data locally during network outages and sync later when connectivity is restored. Finally, the mobile app allows users to log personal information (e.g., height, weight), enabling more personalized and accurate analysis than calculations based on an “average” human model.

Regardless of the transmission path—direct Wi-Fi or a BLE relay via a mobile phone—all data ultimately reaches a Cloud-based Streaming Server, which in our case is hosted on Amazon Web Services (AWS). The data transmission rate varies based on factors such as the device’s chipset bandwidth and data volume. In the experimental setup described here, data points—including user hip position, hip velocity, actuator torque, device ID, and timestamps—were transmitted at a frequency of 10 Hz (i.e., every 100 milliseconds).

### 3.3. Cloud-Based Streaming Server: Amazon Timestream

**Figure 3:**
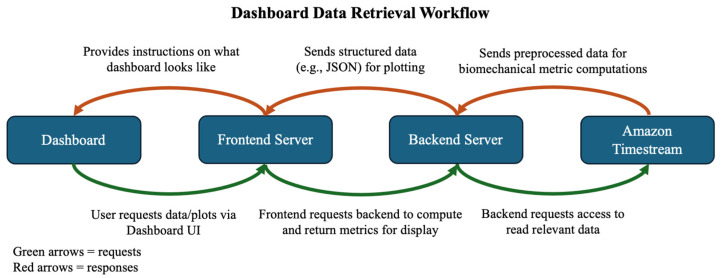
An example of how API’s control information transfer between the Frontend Server, Backend Server, and Cloud-Based Streaming Server (Amazon Timestream).

The Cloud-based Streaming Server—in our case, Amazon Timestream by AWS—acts as a central hub for data ingestion, storage, and preliminary processing. It simultaneously handles incoming data streams from multiple devices, maintaining data integrity by tagging each entry with a timestamp and device ID [27]. The data on this server is accessible via secure Application Programming Interfaces (APIs), which the Backend Server uses to retrieve raw data for computing biomechanical metrics, which are then displayed on the Frontend Server (see Figure 3).

Technically, raw data can be stored on the Cloud-based Streaming Server indefinitely. However, the high volume of data generated by high-frequency sampling can make long-term retention impractical and cost-prohibitive. To address this, data can be filtered or compressed to reduce storage requirements while preserving key information. This approach enables economically feasible long-term storage and supports efficient data retrieval for analysis [28]. For example, storage needs can be substantially reduced by filtering out inactive periods, such as when a user forgets to turn off the device or is taking a break. This can be achieved by first smoothing the data using standard filters and then identifying extended intervals of minimal velocity change to trim the dataset.

### 3.4. Server: Backend

The Backend Server performs the computations that generate meaningful biomechanical and ergonomic metrics. The process is initiated when a user makes a request via the manager’s dashboard on the Frontend Server to view biomechanical data for a specific device and day. This prompt causes the Frontend Server to call the Backend Server’s API. The Backend Server then fetches the relevant raw data from the Cloud-based Streaming Server by querying its API using the specified device ID and timestamp. Using this data, the Backend Server computes key metrics—including spinal compression force, spinal shear force, and back muscle force—by applying physics-based models (as expanded upon in Section 4). These computed metrics are packaged into a structured data file (e.g., JSON) and made accessible to the Frontend Server via the Backend’s API. Finally, the Frontend Server, which hosts the manager’s dashboard, parses this data and presents it in visualizations, such as a plot of spinal compression force over time.

### 3.5. Server: Front End

The Frontend Server delivers the dashboard interface to the user’s browser, typically as JavaScript files that define its layout and functionality. Once loaded, this code runs locally in the browser and makes API requests to the Backend Server to retrieve computed metrics. Through the Frontend’s dashboard, users such as exoskeleton users, clinicians, or developers can securely login, select a device ID and date, and request access to biomechanical data. The Frontend Server then returns both the metrics and plotting instructions for clear and interactive visualization of key outcomes such as spinal compression forces, fatigue estimates, and time spent at-risk measures.

### 3.6. Data Flow Summary

In summary, raw sensor data captured by the exoskeleton is transmitted through a mobile phone, stored in a Cloud-based Streaming Server, transformed into biomechanical metrics in the Backend Server, and visualized through the Frontend Server’s dashboard, which the user interacts with. Figure 2 and Figure 3 illustrate both the layered architecture and the request/response cycle that enables data retrieval to the user.

## 4. Experimental Evaluation

### 4.1. Description of the Exoskeleton Used for Study

**Figure 4:**
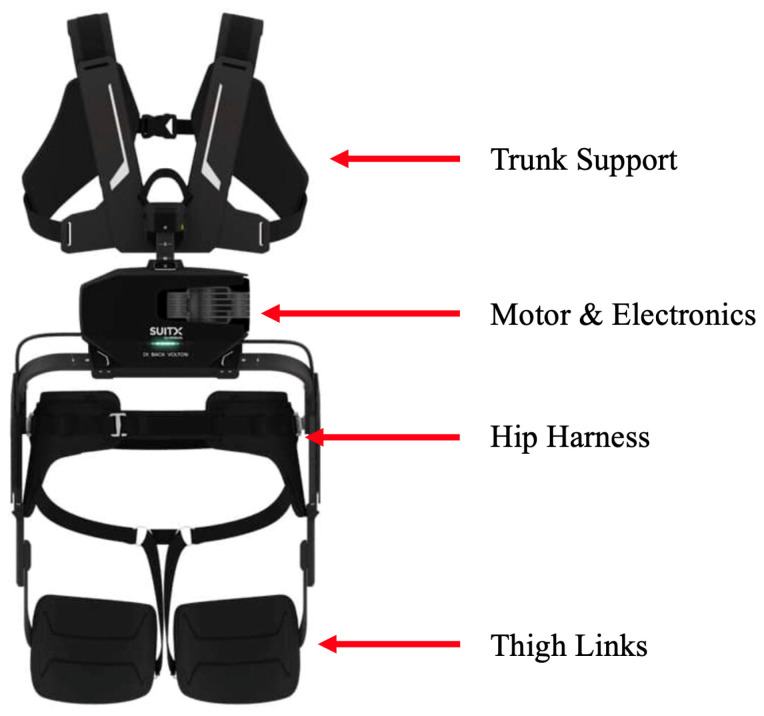
Schematic of the suitX exoskeleton used for evaluation.

**Figure 5:**
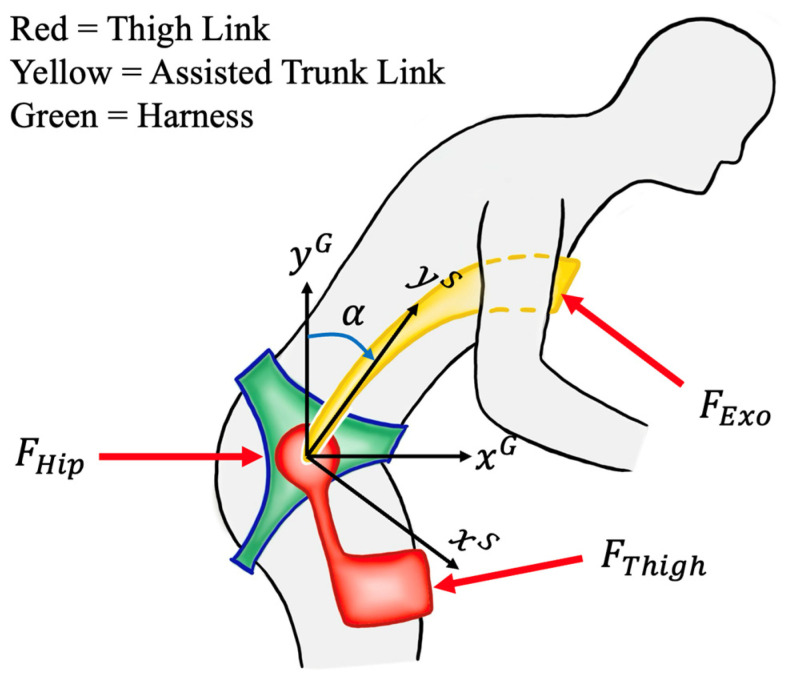
Exoskeleton imposes trunk force $F_{Exo}$, thigh force $F_{Thigh}$ and hip force $F_{Hip}$ on the user. $\left(x^{G},y^{G}\right)$ is the global coordinate frame, and ($x^{S},y^{S})$ is the global frame rotated by α, such that $y^{S}$ points along the approximate direction of the spine.

We used an electrically powered exoskeleton by SuitX (Emeryville, CA, USA) for verification of this evaluation platform. The schematic of the device is shown in Figure 4. The exoskeleton’s frame and straps are worn over the torso and thighs, applying external forces on the chest, thighs, and hip as shown in Figure 5. The design is optimized to provide support perpendicular to the spine, which unloads the back muscles without causing unwanted spinal compression. The electronics, which also contains the microcomputer and BLE module, is held within an electronics box in the frame.

When the user begins to bend forward, the exoskeleton’s sensor detects this change in posture. The system identifies whether the user is in a state of flexion (bending) or extension (lifting). As the user bends forward, creating torque on their lower back, the motor applies a counteracting torque. This provides the user with assistance proportional to their movement, redirecting force from the lower back to the thigh and hip areas, reducing the muscle effort and strain on the lumbar region. When the user returns to an upright position, the motor assists with the extension motion, enabling the user to maintain an ergonomic and healthy posture throughout the lift. More specific information on the controls and mechanical design of the SuitX device can be found in Patent No. US 2025/0248876 A1.

### 4.2. Key Geometric Points of a User for Analysis

**Figure 6:**
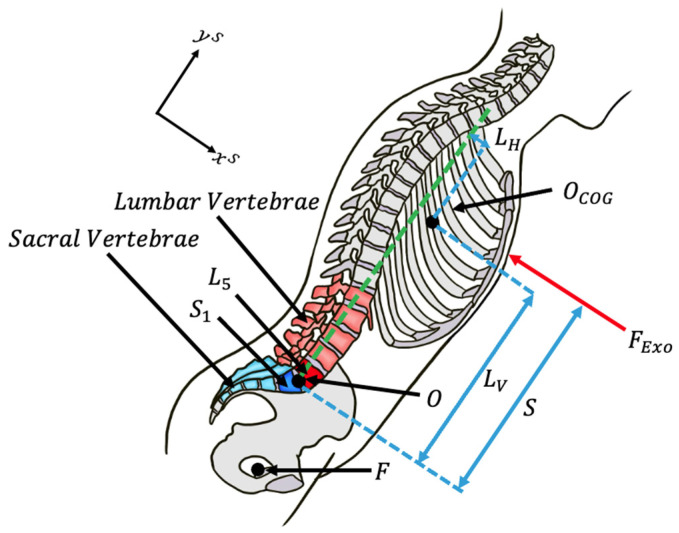
Point O illustrates the lumbosacral joint and Point F illustrates the coxal joint. Point $O_{COG}$ is the center of mass of the upper body, a portion of the exoskeleton weight, and the load being lifted. The dashed line along the spine lies along the $y^{S}$ direction.

The analysis below considers point O to be the lumbosacral joint (i.e., the point where L5 and S1 meet) shown in Figure 6. Lumbar vertebrae, numbered L1 to L5, are situated in the lower back region. The sacrum comprises a group of five vertebrae (S1 to S5) fused together to link the spine to the pelvis, forming a ring known as the pelvic girdle. The L5-S1 region denotes the transitional area between the lumbar spine (L5) and the sacral spine (S1) in the lower back and facilitates the transfer of loads from the spine to the legs and pelvis [29]. Figure 6 also depicts the relationship between point O (L5-S1) and the acetabulum point F—the point where the femur articulates with respect to the pelvis at the coxal joint. A dashed line along the approximate direction of the spine from point O is defined as the direction of the y-axis ($y^{S}$).

We also note that, in many material-handling facilities, the weight of the load being lifted is substantially limited to improve the worker’s quality of work and is, therefore, much smaller than the weight of the user’s trunk, head, and arms (approximately 72% of the body weight). Point $O_{COG}$ is the center of mass of the user’s trunk, head, arms, a portion of the exoskeleton weight, and the load being lifted. This point varies from person to person and within an individual based on their posture. However, in general, the weight of the exoskeleton and the load being lifted is much less than the weight of the user’s trunk.

For simplicity, we assume that the center of mass of the person’s upper body, exoskeleton, and load being lifted is close to the spine, such that $L_{H}$ is close to zero.

### 4.3. Force Analysis of the Exoskeleton User

The following variables define important parameters used for force analysis on the exoskeleton:

$\left(x^{G},y^{G}\right)$: the global coordinate system, where $y^{G}$ points in the opposite direction of the gravity vector.

$\left(x^{S},y^{S}\right)$: the body’s coordinate system, where $y^{S}$ is the approximate direction of the spine.

α: angle between the global frame and the body’s coordinate frame in the sagittal plane quantifying the degree of bending by the user.

$M_{E}$: equivalent mass of the user’s trunk, head, arms, a portion of the exoskeleton, and the load being lifted.

$F_{M}$: muscle forces at the user’s back.

$T_{Exo}$: extension torque from the exoskeleton about point O such that $T_{Exo}=F_{Exo}\cdot S$.

I: moment of inertia of equivalent mass relative to point O.

D: distance between user’s back muscle and point O in $x^{S}$.

$L_{V}$: distance between user’s center of mass and point O in $y^{S}$.

$L_{H}$: distance between the user’s center of mass and point O in the direction orthogonal to the spine ($x^{S}$).

$F_{Exo}$: force imposed on the user’s trunk by the exoskeleton such that $F_{Exo}=$ $\frac{T_{Exo}}{S}$.

S: distance between location of $F_{Exo}$ and point O (along $y^{S}$).

**Figure 7:**
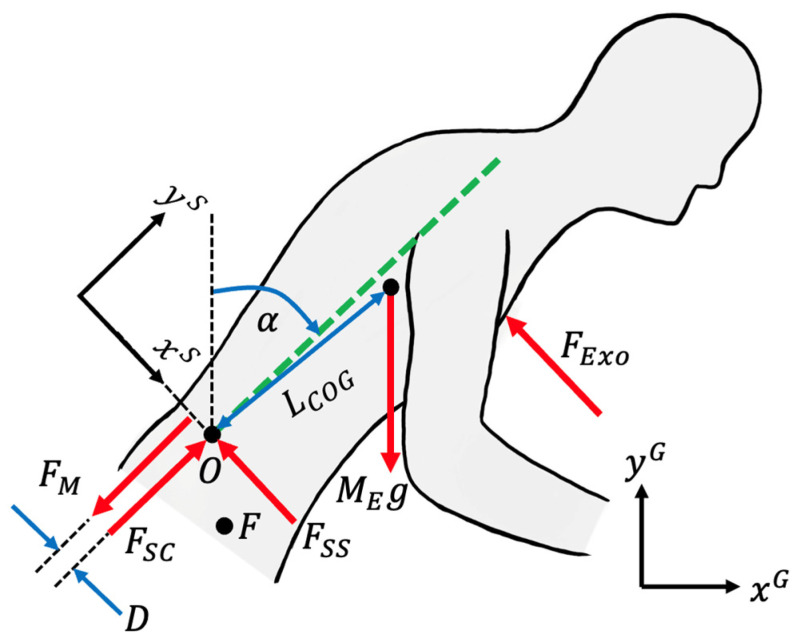
Internal forces in the user’s body with assistance from the exoskeleton.

Figure 7 shows the details of the cross-sectional forces applied to the user’s upper body when an exoskeleton is worn. The spinal compression force, $F_{SC}$, and back muscle forces, $F_{M}$, are assumed to be along the direction of the spine (or in the direction of the $y^{S}$-axis), while the spinal shear force, $F_{SS}$, and force applied by the exoskeleton, $F_{Exo}$, are assumed to lie in the direction perpendicular to the spine (or in the direction of the $x^{S}$-axis).

Employing Newton’s law, muscle forces at the exoskeleton user’s back can be calculated from Equation (1).

(1) $$F_{M}=\frac{1}{D}\left[\left(L_{V}\sin\alpha +L_{H}\cos\alpha \right)M_{E}g-T_{Exo}+I\ddot{\alpha }\right]$$

Since the center of mass is assumed to be close to the spine, the centripetal force from angular rotation acts only in the $y^{S}$ direction, while the tangential acceleration term only acts orthogonally to the spine (in $x^{S}$).

The spinal compression force at point O, $F_{SC}$, can be calculated by Equation (2) (or Equation (3), which is the expanded form).

(2) $$F_{SC}=F_{M}+M_{E}g\cos\left(\alpha \right)-M_{E}L_{COG}{\dot{\alpha }}^{2}$$

(3) $$\begin{array}{c} F_{SC}=\frac{1}{D}\left[\left(L_{V}\sin\alpha +L_{H}\cos\alpha \right)M_{E}g-T_{Exo}+I\ddot{\alpha }\right]+\\ M_{E}g\cos\alpha -M_{E}L_{COG}{\dot{\alpha }}^{2} \end{array}$$

$L_{COG}$ in the equations above is the distance between the trunk center of mass ($O_{COG})$ and point O such that

(4) $$L_{COG}=\sqrt{L_{V}^{2}+L_{H}^{2}}$$

The spinal shear force at point O, $F_{SS}$, can be calculated by Equation (5).

(5) $$F_{SS}=M_{E}g\sin\left(\alpha \right)-\frac{T_{Exo}}{S}-M_{E}L_{COG}\ddot{\alpha }$$

Observing Equations (1) and (2), it becomes evident from looking at the dependencies on $T_{Exo}$ that the exoskeleton alleviates the user’s back muscle forces and spinal compression forces equally by $\frac{T_{Exo}}{D}$ or $F_{Exo}$. Similarly, from Equation (5), the exoskeleton reduces the spinal shear force of the user by $\frac{T_{Exo}}{S}$.

The Newton–Euler free-body-diagram formulation used in Equations (1)–(5) follows methodology established in recent exoskeleton biomechanics literature. Di Natali et al. [30] derived structurally identical momentum equilibrium equations at the L5/S1 joint for a back-support exoskeleton user, and Madinei & Nussbaum applied an optimization-based spinal loading model with an explicit exoskeleton torque input to establish lumbosacral compression and shear forces during repetitive lifting—the same quantities computed here [31]. The use of IMU-derived trunk kinematics ($\alpha ,\dot{\alpha },\ddot{\alpha }$) as model inputs follows the validated approach of Brouwer et al. and Nail-Ulloa et al. [32,33], who demonstrated that inertial motion capture systems provide sufficient accuracy for L5/S1 force estimation during lifting tasks.

### 4.4. Utilizing the ExoMetrix Framework to Display Biomechanical Metrics on a Dashboard

In order to display how an exoskeleton is affecting biomechanical metrics such as back muscle force, spinal compression force, and spinal shear force, developers can employ the ExoMetrix architecture described in Figure 2. The kinematic ($\alpha ,\dot{\alpha },$ and $\ddot{\alpha }$) and actuation data ($T_{Exo}$) are measured through sensors on the exoskeleton and sent via BLE to a paired mobile phone. Personal data, such as height and weight, can be provided by a user in the mobile phone app or on the dashboard user interface. Parameters like height and weight can then be extrapolated to estimate the rest of a user’s bodily parameters: $D,M_{E},L_{V},L_{H},I,$ and S. These datasets are sent to the Cloud-based Streaming Server via Wi-Fi for storage.

When the user (or any other client of the dashboard with authorized access) requests to see summaries of their data for a particular day, the Frontend Server pulls plotting information from the Backend Server. The Backend Server calls on the Timestream API to read relevant data, specifically the kinematic and actuation data logged with their corresponding timestamps. Using this data, the Backend Server utilizes Equations (1), (3) and (5) to compute the back muscle force, spinal compression force, and spinal shear force with and without the exoskeleton’s assistance (inputting $T_{Exo}$ from the data or setting it to zero, respectively). These computation outputs are then returned to the Frontend in an organized manner for plotting.

### 4.5. An Example Dashboard

**Figure 8:**
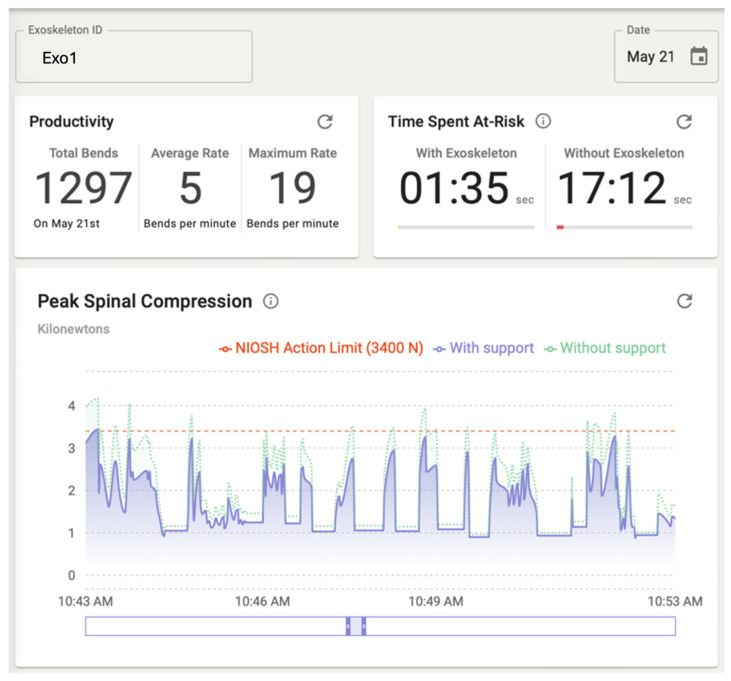
ExoMetrix platform displaying key biomechanical and performance metrics upon device ID and date input.

In the example shown in Figure 8, the dashboard displays summarized metrics for the user corresponding to the device ID “Exo1” and date “21 May”. The panels shown here are productivity, peak spinal compression, and time spent at-risk. Other metrics that can be displayed but are not shown in Figure 8 include the back muscle forces, spine shear force, bend rate over time, and other metrics a user may be interested in.

On the top left is a productivity tab, which displays the total number of bends, the average rate of bending, and the maximum bend rate recorded that day. These metrics are indicative of a user’s workload intensity at a high level.

The bottom plot presents the peak spinal compression forces of the user over time. Since spinal compression force is maximized when the user is at peak hip flexion, Equation (3) is evaluated only at detected flexion peaks (identified using functions like *find_peaks* from the *signal* module in SciPy). The reduction in auxiliary points smooths the plot and allows for more meaningful data to be displayed with less visual clutter. The slider below the plot enables users to interactively adjust the time window by expanding or contracting its range for zooming and by shifting the window to view different time segments.

The purple and green curves represent the spinal compression forces computed with support and without support from the exoskeleton. By observation, a user can visualize how the exoskeleton is helping them by comparing the two curves and see how the exoskeleton consistently reduces peaks compared to no support. At several points, peak spine compression approaches or exceeds the National Institute for Occupational Safety and Health’s (NIOSH) Action Limit of 3400 N, shown as a dashed red line. This Action Limit defines the compressive force threshold at the L5/S1 spinal joint defined in the Revised NIOSH Lifting Equation (RNLE) [34].

The top right panel of Figure 8 displays the time spent at-risk both with and without the support of an exoskeleton. This is quantified as the cumulative duration during which the peak spinal compression exceeded the NIOSH Action Limit of 3400 N. In this example, the exoskeleton significantly reduced the user’s exposure to high-risk spinal loading conditions.

### 4.6. Applications of ExoMetrix

ExoMetrix has broad applications across a diverse range of users and stakeholders. Exoskeleton wearers can view their own biomechanical data in real time, empowering them to understand how the device is affecting their spine loading, fatigue levels, and productivity levels during work. Workplace managers can access aggregated reports that highlight risk exposure trends across teams, enabling data-driven decisions for improving safety practices. Clinicians benefit from longitudinal records that provide concrete, objective evidence for health assessments and rehabilitation planning. Developers and engineers can access specialized datasets to evaluate device performance under real-world conditions.

ExoMetrix can also support ergonomic assessment at the facility scale. By centralizing worker data, ergonomists can monitor workforce-wide trends, identify at-risk individuals, and develop targeted interventions. An AI layer could further augment this capability by surfacing patterns and generating actionable recommendations—for example, identifying periods of elevated risk such as peak warehouse seasons when workers bend more frequently and at higher rates than usual. Capturing such longitudinal data across large worker populations supports both regulatory compliance and the design of safer work environments.

The ExoMetrix system also enables adaptive personalization, allowing exoskeletons to adjust assistance levels dynamically to minimize individual risk exposure. Looking further ahead, we envision extending the platform beyond exoskeleton users—replacing onboard sensors with camera-based pose estimation to derive biomechanical metrics for any worker in a facility, regardless of whether they are wearing a device. As regulatory standards evolve, the framework’s modular architecture makes it straightforward to incorporate updated compliance metrics. Ultimately, by making biomechanical effects transparent and continuously accessible, ExoMetrix builds user trust and lays the groundwork for a new standard in occupational health monitoring.

## 5. Limitations

### 5.1. Model Assumptions

The biomechanical model underlying Equations (1)–(5) relies on simplifying assumptions, including a single-equivalent back muscle representation, planar sagittal motion, and a center of mass assumed to lie close to the spine. These assumptions are consistent with the established literature on occupational spine loading and are sufficient for the platform’s purpose of providing interpretable, real-time biomechanical feedback. ExoMetrix is not intended as a high-fidelity musculoskeletal simulation tool; rather, it provides a flexible computational framework in which the underlying model can be updated or replaced as more accurate formulations become available.

### 5.2. Experimental Validation

The experimental evaluation presented in Section 4 validates the end-to-end functionality of the ExoMetrix platform—confirming that sensor data is correctly acquired, transmitted, stored, and transformed into biomechanical metrics—but does not constitute a standalone validation of the estimated force quantities. A dedicated validation study comparing ExoMetrix outputs under current assumptions against OpenSim simulations or published experimental datasets across a range of subjects and lifting conditions is identified as a primary direction for future work.

### 5.3. Sensor Noise Sensitivity

Like any system involving sensor data collection or fusion, this platform is susceptible to sensor noise. However, the bandwidth of human movements is considerably narrower than typical sensor noise frequencies. Therefore, proper filtering—either implemented by the sensor manufacturer or applied within the platform via classical filtering techniques (e.g., low-pass or Kalman filtering)—should be sufficient to enable reliable kinematic analysis.

### 5.4. Parameter Uncertainty

The platform requires subject-specific parameters that may be difficult to measure and can vary across individuals and over time. This parameter uncertainty remains an important limitation. Nevertheless, we believe that system identification methods can be applied in future work to obtain better estimates of these parameters, thereby improving the accuracy and personalization of the platform without requiring extensive manual measurements.

### 5.5. Latency

Latency is a recognized concern, as large volumes of data must be transferred from the worker’s location to a server and then to a backend server for processing. However, we argue that for the primary purpose of this work—enabling ergonomists to understand how workers have been performing over time and to propose improvements for individuals or entire factories—a moderate amount of latency is not a critical obstacle. As data transfer technologies continue to evolve across global server networks, latency issues will likely diminish. That said, we acknowledge that the sooner the data become available for analysis, the more effective the system will be in providing timely feedback and interventions.

### 5.6. Scalability

Scalability presents another limitation, depending on server capacity and the specific architecture constraints of the system. For very large numbers of workers, the current design may not scale seamlessly. However, this challenge can be mitigated relatively easily. The current data collection rate is 10 Hz, which is significantly faster than necessary for ergonomic and economic analysis. By reducing the collection frequency (e.g., to several samples per second), the system can accommodate a much larger worker population with minimal loss of meaningful information. Further optimization of the cloud architecture will also help address scalability in the near future.

### 5.7. Model Reliability and Future Outlook

Our metric outcomes are only as reliable as the underlying models and the data processing and filtering methods used. While a degree of uncertainty will always be inherent, the applications and potential of this framework in real-world settings are significant, as described in Section 4.6. It is our belief that the precision in modeling will improve upon more widespread use of machine learning, which improves the reliability of the method described here.

### 5.8. Data Security, Privacy, and Ethics

Data security and privacy also remain important considerations, though they are beyond the scope of this paper. The ethical use of this platform must be emphasized. We intend for this data to enhance the work life and health of exoskeleton users by providing them access to meaningful feedback, not to be misused by employers for purposes such as penalizing or dismissing workers at higher risk of injury.

## 6. Future Work

A primary direction for future work is a dedicated validation study of the biomechanical force estimates produced by ExoMetrix. This will involve comparing the platform’s outputs—back muscle force, spinal compression force, and spinal shear force—against reference measurements from established musculoskeletal simulation tools such as OpenSim and AnyBody, as well as against published experimental datasets from instrumented lifting trials. Such a study will also systematically characterize the sensitivity of model outputs to inter-subject variability in anthropometric parameters (e.g., body mass, trunk height, back muscle moment arm) and assess reliability across repeated trials and varying task conditions. This validation effort will establish the accuracy bounds within which ExoMetrix outputs should be interpreted and will inform future refinements to the underlying biomechanical problem—including the potential integration of machine learning techniques to improve personalization and robustness across users and work environments.

Another direction for future work is the development of large-scale, longitudinal datasets of human–exoskeleton interaction enabled by the ExoMetrix framework. By aggregating data across users, tasks, and environments, ExoMetrix can support systematic benchmarking of exoskeleton performance across device architectures and real-world conditions, addressing the current lack of standardized evaluation in the field.

These datasets also enable the establishment of population-level biomechanical norms, providing insight into typical loading patterns and variability in movement strategies across work populations. Such information can inform improved ergonomic guidelines, safety thresholds, and design requirements for wearable robotic systems. Further, machine learning techniques can be used to improve model accuracy, account for inter-subject variability, and capture complex behaviors that are difficult to model analytically.

Finally, integrating these datasets with data-driven models can enable predictive, personalized applications. Combining physics-based models with machine learning may improve robustness across users, support adaptive assistance strategies, and help identify early signs of injury risk from long-term data.

## 7. Conclusions

This work presents the ExoMetrix system as a prototype framework for real-time model-based evaluation of human–exoskeleton interaction. By integrating sensing, cloud-based data infrastructure, and backend computation, ExoMetrix demonstrates how onboard device signals can be transformed into meaningful biomechanical metrics, including spinal compression forces, spinal shear forces, and back muscle forces. In doing so, it addresses the limitations of conventional lab-based evaluation methods, such as surface electromyography, by offering a scalable, field-deployable solution that directly quantifies internal loading conditions. This framework has the potential to support device personalization, long-term monitoring, and future compliance-oriented evaluation workflows. Real-time biomechanical dashboards may become an important feature of future wearable assistive technologies. Secure and standardized evaluation frameworks will be important for broader industrial adoption.

## Data Availability

The raw data supporting the conclusions of this article will be made available by the authors on request.
